# Veteran Status and Old Age Depression: A Life Course Epidemiology Study

**DOI:** 10.1093/geroni/igaa057.2184

**Published:** 2020-12-16

**Authors:** Stephen Frochen, Connor Sheehan, Jennifer Ailshire

**Affiliations:** 1 University of Southern California, Los Angeles, California, United States; 2 Arizona State University, Tempe, Arizona, United States

## Abstract

Military service and exposure to war may influence the development of depression, leading to disparities in the condition among veterans and non-veterans. This study included 10,512 older men from the 1996 to 2014 waves of the Health and Retirement Study (HRS). We estimated Center for Epidemiology Depression (CESD) score trajectories among veterans and non-veterans and veterans of different war cohorts in growth curve models, controlling for early, mid, and late life characteristics. CESD score trajectories were lower among veterans and war veterans than non-veterans and non-war veterans, respectively. The highest levels of depression were among war veterans who served in more than one war. Veterans demonstrated lower levels of depression than non-veterans, calling into question the health advantage of veterans and selection mechanisms into the military and out of HRS. Multiple war veterans showed the highest levels of depression, representing the greatest mental health threat to veterans in the study. Part of a symposium sponsored by the Aging Veterans: Effects of Military Service across the Life Course Interest Group.

